# Characterizing Host Inflammatory Bioprofiles in People With Postacute Sequelae SARS‐CoV‐2 Infection (PASC)

**DOI:** 10.1155/jimr/6436047

**Published:** 2026-09-29

**Authors:** Ninon Marmont, Alejandro Abner Garcia Leon, Grace Kenny, Andrew Conroy, Colette Gaillard, Fiorina Rigonat, Polina Smolovyk, Eoghan de Barra, Eoin Feeney, Kathleen McCann, Mary Horgan, Obada Yousif, Corinna Sadlier, Aoife G. Cotter, Jane A. O’Halloran, Stefano Savinelli, Patrick W. G. Mallon

**Affiliations:** ^1^ Infection Research Ireland at UCD, School of Medicine, University College Dublin, Dublin, Ireland, ucd.ie; ^2^ Centre International de Recherche en Infectiologie (CIRI), INSERM U1111, Unité Mixte de Recherche 5308, Ecole Normale Supérieure de Lyon, Centre national de la recherche scientifique, Lyon, France, cnrs.fr; ^3^ St Vincent’s University Hospital, Dublin, Ireland, stvincents.ie; ^4^ Department of International Health and Tropical Medicine, Royal College of Surgeons in Ireland, Dublin, Ireland, rcsi.ie; ^5^ Beaumont Hospital, Dublin 9, Beaumont, Ireland, beaumont.ie; ^6^ Mater Misericordiae University Hospital, Dublin, Ireland, mater.ie; ^7^ Wexford General Hospital, Wexford, Ireland; ^8^ Department of Infectious Diseases, Cork University Hospital, Wilton, Cork, Ireland, ucc.ie

**Keywords:** biomarkers, bioprofiling, clusters analysis, PASC, postacute sequelae of SARS-CoV-2 infection, SARS-CoV-2

## Abstract

Postacute sequelae of COVID‐19 or “PASC” is a common but heterogeneous condition associated with significant morbidity. Although its pathogenesis remains poorly understood, proposed underlying mechanisms include altered host immune and inflammatory responses and altered coagulation. We measured 57 circulating plasma biomarkers mapping eight biological pathways (axonal injury, inflammation, antiviral immune responses, immune regulation/innate immune activation, tissue damage and repair, endothelial dysfunction, coagulation, and microbial translocation) in a cohort of 356 participants attending with PASC. Using principal component analysis (PCA) and unsupervised clustering, we identified six distinct inflammatory clusters within this PASC cohort, three of which were characterized by elevated biomarkers related to tissue damage and repair, coagulation, axonal injury, and antiviral immune responses. These three “highly inflamed” clusters were associated with reported cardiovascular and respiratory symptoms in fully adjusted analysis. These data suggest a link between distinct host immune responses and different clinical PASC phenotypes.

## 1. Introduction

The emergence of the SARS‐CoV‐2 pandemic in humans has resulted in a number of distinct clinical manifestations, one of the most notable of which is the postacute sequelae of COVID‐19, commonly known as PASC. For the majority of affected people, SARS‐CoV‐2 infection generally induces a mild to moderate illness, limited to several days or weeks [1]. However, some continue to experience long‐term symptoms or even develop new symptoms beyond this acute phase, including pulmonary, cardiovascular, nervous system, and psychological symptoms. A meta‐analysis of 429 studies estimated a global prevalence of PASC of ~36% among people who have had confirmed COVID [2]. This diverse collection of clinical presentations is considered under the umbrella of PASC [3–6]. Initially, the absence of a consensus clinical case definition presented a challenge to advancing research and management of affected individuals. In response to this challenge, the World Health Organization (WHO) defined PASC as the continuation or development of new symptoms 3 months after the initial SARS‐CoV‐2 infection, with symptoms persisting for at least 2 months with no other explanation. An estimated 200 million people have developed PASC, and in some cases, the persistence of symptoms can extend beyond a few years [1].

Likely arising from the diversity of clinical presentations and lack of consensus case definition, it is not surprising that studies on PASC are heterogeneous. Over 200 different symptoms have been reported, with common symptoms including fatigue, breathlessness, and cognitive dysfunction, with significant functional impact [4, 5, 7]. A number of groups have attempted to subclassify PASC based on commonly presenting symptoms, with cardiorespiratory, musculoskeletal, and neurological phenotypes suggested [8]. The pathogenesis of PASC also remains poorly understood, with a number of potential pathogenic mechanisms proposed, including viral persistence of SARS‐CoV‐2 with central nervous system involvement as an explanation for the multiple reported neurological symptoms, including cognitive dysfunction (“brain fog”), headaches, and dysautonomia [8]. Multiple studies have also demonstrated persistent postinfectious immune dysregulation in both acute COVID‐19 and PASC with high levels of several proteins involved in inflammation, antiviral immunity, tissue repair, microbial translocation, coagulation, innate immune activation, and immune regulation pathways—showing notably a persistent complement dysregulation [6, 9–18]. These also include sustained activation of proinflammatory monocytes, reduced naïve T‐cell populations with increased markers of T‐cell exhaustion, and an imbalanced cytokine expression, involving elevated IL‐6, TNF‐α, and other inflammatory mediators. Also, the classical complement pathway also appears to be affected in PASC, contributing to persistent activation of the immune system and prolonged symptoms. Together, these findings support the notion that chronic, low‐grade inflammation is maintained by combined alterations in innate and adaptive immunity [19–23]. Additionally, direct SARS‐CoV‐2 infection of endocrine glands, leading to alteration of the hypothalamic‐pituitary‐adrenal axis, has also been proposed, with hypoadrenalism, hypothyroidism, hyperthyroidism, and thyroiditis all reported [20].

Identifying the likely multiple underlying biological mechanisms driving PASC is crucial to enable subclassification of affected individuals to assist with appropriate, targeted diagnostic and treatment strategies. More specifically, there is very limited research clearly defining circulating biomarkers and their association with PASC.

Our research group has previously identified distinct inflammatory phenotypes in people with HIV that were linked to long‐term clinical outcomes, such as cardiovascular disease [24]. In this study, we used a similar approach to characterize the inflammatory profiles of participants with PASC by quantifying levels of circulating biomarkers mapped to inflammatory and coagulation pathways implicated in the host response to SARS‐CoV‐2 infection and to explore how these inflammatory phenotypes are associated with major clinical phenotypes observed in PASC.

## 2. Materials and Methods

### 2.1. Data Collection

The All‐Ireland Infectious Diseases (AIID) cohort is a multicenter prospective cohort study recruiting people who access infectious disease services at multiple hospitals in Ireland (PMID: 35265728). Over 5000 participants have provided written informed consent for use of their routine clinical data and biobanked blood and respiratory samples, providing a substantial and diverse cohort for medical research. For the purpose of this study, we included AIID participants with symptoms of PASC for at least 4 weeks after a confirmed diagnosis of COVID‐19 (documented positive SARS‐CoV‐2 PCR on respiratory specimens) [4]. Participants were recruited at a time when wild‐type SARS‐CoV‐2, alpha or delta variants were circulating (December 2020–February 2022). For this study (RS20‐033), we selected samples collected within a narrow temporal window of 4–10 weeks postinfection to minimize variability related to time since acute illness. Samples collected more than 10 weeks postinfection were excluded from the analysis. Clinical data were recorded on an electronic case report form (REDCap). Symptom assessment was conducted using self‐reported questionnaires assessing 12 common PASC‐related symptoms: fatigue, SOB, chest pain, palpitations, poor concentration, joint pain, myalgia, headache, gastrointestinal symptoms, dizziness, cough, and anosmia, with clinical confirmation performed by a clinician (Grace Kenny). The symptom evaluation methodology has been previously validated and described in detail elsewhere [4]. Medication data (corticosteroids, immunomodulatory therapies, and anticoagulants) were not systematically recorded, which represents a limitation as these may influence biomarker profiles. Future PASC biomapping registries should systematically track active medication logs to control for these important pharmacological variables. Participants with incomplete symptom data were excluded from the analysis to ensure data completeness for clustering algorithms. Only participants with complete biomarker and clinical symptom profiles were retained for the final cohort. Of 417 participants initially enrolled, 61 (14.6%) were excluded prior to analysis: 29 (7.0%) due to incomplete symptom questionnaire data (>20% missing items) and 32 (7.7%) due to inadequate timing of blood sampling relative to infection date or missing biomarker measurements. The final analytic cohort comprised 356 participants with complete data for all symptom items and biomarker measurements. In addition, participants no longer attending clinic were also followed up clinically by phone to assess recovery using a brief questionnaire. Recovery was defined as the disappearance of symptoms and a return to work/school.

### 2.2. Laboratory Analysis

Stored plasma samples from EDTA tubes were used to measure 57 soluble biomarkers (Table S1) using quantitative immunoassay platforms; Luminex (Catalog Number LXSAHM‐30 and LXSAHM‐09, Luminex Corporation, Austin, TX, USA) and MSD (Cat# K15067M‐2, Meso Scales Diagnostics, Rockville, MD, USA), along with two proteins by single plex measured on SpectraMax iD3: histamine (ENZ‐KIT140A‐0001, Enzo Life Sciences, Switzerland) and β‐D‐Glucan (HUFI01365, Assay Genie, Ireland). Biomarkers were specifically selected from eight biological pathways implicated in PASC: axonal injury, inflammation, antiviral immunity, immune regulation/innate immune activation, tissue repair, endothelial dysfunction, coagulation, and microbial translocation (Figure S1A–H, Tables S1, S3A–H). Selection prioritized markers with established evidence in neuroimmune and vascular dysfunction given their central role in PASC pathogenesis.

Each assay plate included a 7‐point standard curve alongside biological controls. To ensure accuracy and reproducibility, all samples, controls, and standard curves were analyzed in technical duplicates for all plates.

### 2.3. Statistical Analysis

As biomarker data were not normally distributed (assessed by Anderson‐Darling, Shapiro–Wilk, D’Agostino, Pearson, and Kolmogorov–Smirnov analyses), biomarker concentrations were log transformed and scaled (standardized) to ensure biomarkers with larger intrinsic variation did not dominate further analysis.

The analysis was conducted using two stages. The first stage explored differences in individual biomarkers between participants, grouped into three previously described clinical phenotypes [4]; “Musculoskeletal” phenotype, characterized by joint pain and myalgia and a high overall number of symptoms, “Cardiorespiratory” phenotype, characterized by dyspnea and chest pain, and finally a “Less Symptomatic” phenotype, mainly characterized by fatigue.

In the second stage analysis, biomarker data were modeled into clusters. Principal component analysis (PCA) was first performed to explore the structure of the biomarker dataset and for dimensionality assessment and visualization. Unsupervised clustering was then performed using K‐means (with Euclidean distance) directly on the full standardized biomarker data. The optimal number of clusters was determined using multiple complementary approaches, including the elbow method, a silhouette analysis, a gap statistic, and NbClust. Cluster stability was further assessed using bootstrap resampling with Jaccard similarity indices. Resulting clustering data were represented by creating a heatmap (using the “ComplexHeatmap” R package) [25]. We then compared the prevalence of specific clinical characteristics and reported symptoms of PASC across clusters.

Categorical and continuous variables were compared by chi‐square and Kruskal–Wallis tests (group >2), respectively, followed by Dunn’s multiple comparison tests. We used logistic and multivariate regression to investigate relationships between clusters and covariates, adjusting for age, sex at birth, BMI, ethnicity, smoking status, hypertension, and diabetes. Due to the small sample size in Cluster 1 (*n* = 4), we identified potential statistical instability concerns for subsequent regression analyses, which could result in unreliable parameter estimates and excessively wide confidence intervals (CIs). To ensure robust statistical inference, Cluster 1 was merged with Cluster 2 based on their similar low‐inflammatory profiles, creating a more statistically stable reference category for comparative analyses. All other clusters were retained in their original configuration as they met the minimum sample size requirements for stable statistical estimation. Data were represented using heatmaps and forest plots indicating the odds ratio (95% CI).

The association of the clinical phenotypes and recovery from PASC was explored using binary logistic regression, with recovery status (Yes or No) as the dependent variable, adjusting for demographic factors.

In exploratory analyses, a correlation was sought between the biomarkers overexpressed in the three inflammatory clusters and the different components of the complement system using STRINGdb software.

All statistical analyses were performed using GraphPad Prism (GraphPad Software 8.3.0, La Jolla, USA), RStudio Team (Version 2023, PBC, Boston, MA) and Statistical Package for the Social Sciences (IBM SPSS Statistics, Version 27) software. Data are presented as median (IQR) unless stated, and a *p*‐value ≤0.05 was considered statistically significant.

## 3. Results

A total of 356 participants were included in this analysis. The median (IQR) age was 44 (35–53) years, median BMI was 27.5 (24.2–32.8) kg/m^2^, 74.4% were female, and 85.2% were Caucasian. Table 1 summarizes the participant’s characteristics. Participants were classified according to predetermined symptom‐derived phenotypes as cardiorespiratory (*N* = 136, 38.2%), musculoskeletal (*N* = 88, 23.8%) and less symptomatic (*N* = 134, 37.6%). These phenotypes were based on symptoms reported by participants alongside a clinical assessment. We examined whether the sex distribution varied across the identified clusters. No significant differences in sex distribution across clusters were observed (*p* = 0.7), with female participants comprising the majority across all clusters, consistent with the overall cohort composition (74.4% female). Vaccination status was also assessed, with no significant differences observed across clusters (18 patients, 5.1%).

**Table 1:** Participant demographics.

Characteristic	Overall cohort *n* = (356)^a^	Cluster 1 (*n* = 4)^a^	Cluster 2 (*n* = 108)^a^	Cluster 3 (*n* = 140)^a^	Cluster 4 (*n* = 42)^a^	Cluster 5 (*n* = 43)^a^	Cluster 6 (*n* = 19)^a^	*p*‐Value^b^
Age (years)	44.0 (35.0, 53.0)	40.5 (35.0, 45.3)	39.0 (29.0, 48.3)	44.0 (37.0, 50.3)	57.0 (49.0, 64.8)	46.0 (31.5, 56.5)	49.0 (39.0, 59.0)	**<0.001**
Sex at birth (F)	265/355 (75%)	2/4 (50%)	80/108 (74%)	106/140 (76%)	29/42 (69%)	34/42 (81%)	14/19 (74%)	0.7
Ethnicity (Caucasian)	301/353 (85%)	3/4 (75%)	94/108 (87%)	111/138 (80%)	40/42 (95%)	37/42 (88%)	16/19 (84%)	0.2
BMI	27.5 (24.2, 32.8)	24.0 (22.8, 27.0)	25.9 (23.6, 30.3)	27.2 (24.3, 32.7)	30.6 (28.1, 33.6)	28.3 (24.5, 35.1)	32.1 (26.3, 34.6)	**0.003**
Smoking	102/348 (29%)	0/4 (0%)	29/107 (27%)	36/139 (26%)	14/41 (34%)	19/42 (45%)	4/15 (27%)	0.2
Alcohol consumption	105/333 (32%)	0/4 (0%)	29/100 (29%)	46/131 (35%)	12/42 (29%)	10/40 (25%)	8/16 (50%)	0.3
Presence of symptoms								
Fatigue	295/355 (83%)	4/4 (100%)	87/108 (81%)	120/140 (86%)	31/42 (74%)	35/42 (83%)	18/19 (95%)	0.3
SOB	245/355 (69%)	3/4 (75%)	65/108 (60%)	100/140 (71%)	28/42 (67%)	32/42 (76%)	17/19 (89%)	0.091
Chest pain	124/355 (35%)	1/4 (25%)	42/108 (39%)	49/140 (35%)	7/42 (17%)	17/42 (40%)	8/19 (42%)	0.12
Palpitations	88/355 (25%)	2/4 (50%)	37/108 (34%)	35/140 (25%)	4/42 (9.5%)	5/42 (12%)	5/19 (26%)	**0.005**
Poor concentration	145/355 (41%)	3/4 (75%)	48/108 (44%)	60/140 (43%)	17/42 (40%)	8/42 (19%)	9/19 (47%)	**0.032**
Joint pain	99/355 (28%)	2/4 (50%)	29/108 (27%)	45/140 (32%)	9/42 (21%)	9/42 (21%)	5/19 (26%)	0.5
Myalgia	74/355 (21%)	2/4 (50%)	20/108 (19%)	36/140 (26%)	6/42 (14%)	7/42 (17%)	3/19 (16%)	0.3
Headache	94/355 (26%)	1/4 (25%)	40/108 (37%)	32/140 (23%)	9/42 (21%)	8/42 (19%)	4/19 (21%)	0.11
Gastrointestinal	42/355 (12%)	0/4 (0%)	12/108 (11%)	16/140 (11%)	2/42 (4.8%)	6/42 (14%)	6/19 (32%)	0.12
Dizzy	51/355 (14%)	1/4 (25%)	16/108 (15%)	22/140 (16%)	6/42 (14%)	5/42 (12%)	1/19 (5.3%)	0.8
Cough	59/355 (17%)	1/4 (25%)	16/108 (15%)	24/140 (17%)	6/42 (14%)	6/42 (14%)	6/19 (32%)	0.5
Anosmia	41/355 (12%)	0/4 (0%)	10/108 (9.3%)	19/140 (14%)	4/42 (9.5%)	6/42 (14%)	2/19 (11%)	0.9
Time from symptom onset	23.0 (13.9, 36.4)	13.6 (9.4, 24.5)	25.1 (14.4, 45.4)	25.1 (15.3, 36.1)	17.1 (9.4, 27.0)	20.1 (14.7, 37.5)	18.7 (15.1, 27.2)	0.056
WHO severity								
Critical	12/353 (3.4%)	0/4 (0%)	2/108 (1.9%)	7/138 (5.1%)	1/42 (2.4%)	1/42 (2.4%)	1/19 (5.3%)	
Mild	293/353 (83%)	3/4 (75%)	99/108 (92%)	116/138 (84%)	28/42 (67%)	35/42 (83%)	12/19 (63%)	
Moderate	35/353 (9.9%)	1/4 (25%)	6/108 (5.6%)	11/138 (8.0%)	7/42 (17%)	5/42 (12%)	5/19 (26%)	
Severe	13/353 (3.7%)	0/4 (0%)	1/108 (0.9%)	4/138 (2.9%)	6/42 (14%)	1/42 (2.4%)	1/19 (5.3%)	
Comorbidities								
Hypertension	64/346 (18%)	1/4 (25%)	10/107 (9.3%)	24/136 (18%)	16/41 (39%)	9/39 (23%)	4/19 (21%)	**0.002**
Diabetes	19/346 (5.5%)	1/4 (25%)	2/107 (1.9%)	4/136 (2.9%)	9/41 (22%)	1/39 (2.6%)	2/19 (11%)	**<0.001**
Obesity	56/346 (16%)	0/4 (0%)	11/107 (10%)	21/136 (15%)	13/41 (32%)	7/39 (18%)	4/19 (21%)	0.057
Liver disease	9/346 (2.6%)	0/4 (0%)	3/107 (2.8%)	0/136 (0%)	2/41 (4.9%)	3/39 (7.7%)	1/19 (5.3%)	**0.025**
Respiratory disease	63/346 (18%)	1/4 (25%)	12/107 (11%)	17/136 (13%)	13/41 (32%)	11/39 (28%)	9/19 (47%)	**<0.001**
Cardiac disease	16/346 (4.6%)	0/4 (0%)	3/107 (2.8%)	4/136 (2.9%)	3/41 (7.3%)	3/39 (7.7%)	3/19 (16%)	0.1
Malignancy	10/346 (2.9%)	0/4 (0%)	2/107 (1.9%)	3/136 (2.2%)	5/41 (12%)	0/39 (0%)	0/19 (0%)	0.054
Immunosuppression (including HIV)	4/346 (1.2%)	0/4 (0%)	0/107 (0%)	0/136 (0%)	2/41 (4.9%)	1/39 (2.6%)	1/19 (5.3%)	**0.015**
Neurological disease	9/346 (2.6%)	0/4 (0%)	5/107 (4.7%)	2/136 (1.5%)	1/41 (2.4%)	1/39 (2.6%)	0/19 (0%)	0.6
Other comorbidity	75/346 (22%)	2/4 (50%)	14/107 (13%)	29/136 (21%)	17/41 (41%)	5/39 (13%)	8/19 (42%)	**<0.001**
SARS‐CoV‐2 variant								
VOC	137/355 (39%)	1/4 (25%)	32/108 (30%)	50/140 (36%)	31/42 (74%)	20/42 (48%)	3/19 (16%)	
WT	218/355 (61%)	3/4 (75%)	76/108 (70%)	90/140 (64%)	11/42 (26%)	22/42 (52%)	16/19 (84%)	
Missing	1	0	0	0	0	1	0	
Recovery assessment								
No	160/356 (45%)	2/4 (50%)	48/108 (44%)	61/140 (44%)	22/42 (52%)	17/43 (40%)	10/19 (53%)	
Yes	196/356 (55%)	2/4 (50%)	60/108 (56%)	79/140 (56%)	20/42 (48%)	26/43 (60%)	9/19 (47%)	

*Note:* The values in bold correspond to statistically significant *p*‐values.

^a^ *n*/*N* (%); median (IQR).

^b^Fisher’s exact test; Kruskal–Wallis rank sum test.

The first‐stage analysis compared the levels of 57 circulating biomarkers between these three predetermined symptom‐derived clusters. The objective was to see whether there were differences in expression of specific biomarkers between participants grouped into each predetermined symptom‐derived cluster and to assess whether specific pathways were associated with the predetermined symptom‐derived clusters. We did not find significant differences in individual biomarker levels between the three symptom‐derived clusters (Figure 1). Additionally, we found no association between cluster membership and individual biomarker levels in regression analyses adjusted for demographic factors (age, sex at birth, ethnicity, and BMI). Taken together, these findings indicate that symptom‐based classification alone does not capture the underlying inflammatory heterogeneity of PASC.

**Figure 1:**
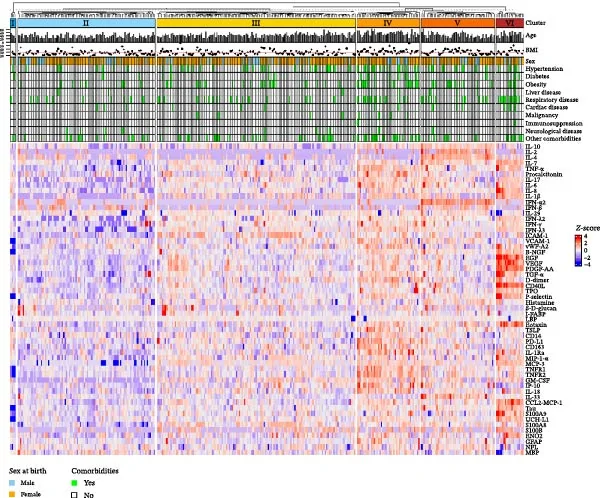
Heatmap of the six distinct clusters and plots showing proteins specifically expressed and organized by pathways. Heatmap demonstrating unsupervised clustering of the expression of each biomarker for each patient (overall cohort *n* = 356; 57 biomarkers measured). Cluster 1 (*n* = 4), Cluster 2 (*n* = 108), Cluster 3 (*n* = 140), Cluster 4 (*n* = 42), Cluster 5 (*n* = 43), and Cluster 6 (*n* = 19). Data represent single measurements from *n* = 356 participants. No technical replicates were performed. Categorical variables were compared using chi‐square tests, continuous variables using Kruskal–Wallis tests followed by Dunn’s multiple comparison tests. K‐means clustering with Euclidean distance on standardized data. Optimal cluster number determined using the elbow method, silhouette analysis, gap statistic, and NbClust. *p*‐Values < 0.05 were considered statistically significant. Cluster stability assessed using bootstrap resampling with Jaccard similarity indices. The cluster with the least expression is shown on the left and with the highest on the right. For the more inflamed clusters, specific pathways really expressed are highlighted by the plot in Figure 2.

**Figure 2:**
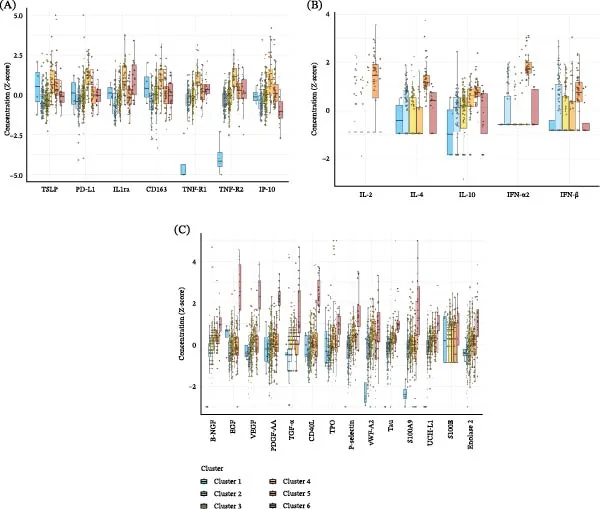
Key biomarkers signatures by cluster. (A) Cluster 4 (*n* = 42), (B) Cluster 5 (*n* = 43), (C) Cluster 6 (*n* = 19). Each data point represents a single biomarker measurement from individual participants (Cluster 4 [*n* = 42], Cluster 5 [*n* = 43], and Cluster 6 [*n* = 19]). No technical replicates were performed. Statistical significance was assessed using Kruskal–Wallis tests followed by Dunn’s multiple comparison tests with significance set at *p* < 0.05.

In the second stage analysis, unsupervised clustering of the 57 biomarkers revealed six clusters, characterized by distinct patterns of inflammation (Figure 1). Within these, clusters 4, 5, and 6 showed relatively higher levels of inflammatory biomarkers, cluster 3 showed intermediate inflammatory responses, and clusters 1 and 2 showed relatively lower levels of inflammatory biomarkers overall. As detailed below, these biomarker‐derived clusters were subsequently found to associate with specific clinical manifestations, underscoring the complementary and nonredundant value of a biomarker‐first approach relative to symptom‐based phenotyping alone.

Clusters 1–3 represented 70.8% of the overall analyzed cohort, with Cluster 1 including 4 participants, Cluster 2 108 participants, and Cluster 3 140 participants. Overall, participants in these clusters had lower BMI, younger age, and fewer comorbidities than participants in clusters 4, 5, and 6 (Table 1). We combined these clusters into one overall comparator group.

Of the three clusters characterized by higher levels of inflammation, Cluster 4 (*n* = 42 [11.8%]) was characterized by higher biomarkers of innate immune activation (TSLP, PD.L1, IL‐1Ra, CD163, TNFR1, TNFR2, IP‐10, Figure 2A), and had higher BMI (30.6 [28.1–33.6] kg/m^2^), were older (57 [49–64.8] years), and had higher prevalence of comorbidities such as hypertension (39%), obesity (32%) and respiratory disease (32%), compared to the comparator group.

Cluster 5 participants (*n* = 43 [12.1%]) had higher biomarkers mapping to anti‐inflammatory cytokines (IL‐2, IL‐4, IL‐10) and antiviral immune pathways (Interferon‐α2 [IFN‐α2], IFN‐β, Figure 2B), were relatively younger (46 [31.5–56.5] years) but also had higher prevalence of comorbidities, notably respiratory disease (28%), hypertension (23%) and obesity (18%), compared to the comparator group.

Cluster 6 (*n* = 19, 5.3%) was notable for higher levels of biomarkers mapping to tissue damage biomarkers (VEGF, EGF, PDGF, TGF‐α, β‐NGF), coagulation (CD40L, P‐selectin, TPO, vWF), and axonal injury (S100A9, S100B, Tau, ENO2, UCH‐L1, Figure 2C). It should be noted, however, that several biomarkers within the axonal injury pathway are not entirely CNS‐specific and may also reflect systemic inflammation or vascular injury; this signature should therefore be interpreted as suggestive rather than definitive evidence of neuronal. Interestingly, of the participants in this cluster, most (58%) belonged to the previously classified “Cardiorespiratory” clinical group and the majority (84%) were infected with SARS‐CoV‐2 in the first wave of the pandemic, when “wild type” SARS‐CoV‐2 was the predominant circulating virus. Also, Cluster 6 showed a numerically higher prevalence of poor concentration compared to other clusters, although this did not reach statistical significance in adjusted analyses. Participants in this cluster also had the highest BMI (32.1 [26.3–34.6] kg/m^2^) and high prevalence of comorbidities such as respiratory disease (47%), hypertension (21%), and obesity (21%) (Table 1).

Nominal logistic regression was used to explore the association between inflammatory clusters and specific clinical symptoms associated with PASC, using the less inflamed clusters (combined clusters 1 and 2) as a reference category (Figure 3), adjusting for demographic characteristics (age, sex at birth, ethnicity, BMI, smoking status, hypertension, and diabetes). We observed that the prevalence of cardiac and respiratory symptoms of PASC was strongly associated with Cluster 6 (adjusted odds ratio 5.6 [95% CI 1.1, 28.4] and 5.4 [95% CI 1.7, 17.7], respectively). In contrast, although we observed associations between clusters 4 and 5 with higher odds of presenting with respiratory symptoms, this association did not remain statistically significant after adjustment for demographic characteristics.

**Figure 3:**
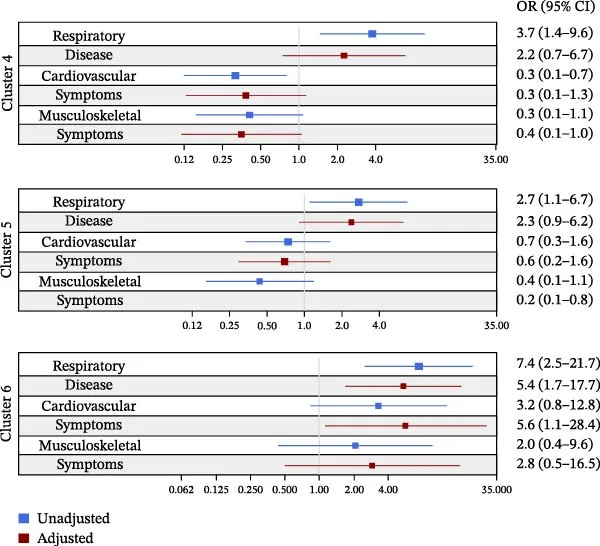
Multivariate model adjusted for age, sex at birth, ethnicity, BMI, smoking status, hypertension, and diabetes. Multivariate analysis, using the less inflamed clusters 1 and 2 as a reference category, and adjusting for demographic characteristics.

Correlation analysis between symptoms and individual biomarkers (independent of cluster membership) revealed distinct patterns of inflammation linked to specific symptoms (Figure S2). The respiratory symptoms of shortness of breath (SOB) and cough were associated with significantly higher levels of a number of biomarkers, including inflammation (TNF‐α, IL‐1β), tissue repair (EGF, PDGFAA, VEGF), coagulation (CD40L), immune regulation (CD14, IL‐18), and axonal injury (S100A8) pathways. In contrast, cardiac symptoms of palpitations and chest pain correlated with relatively lower levels of inflammatory biomarkers belonging to antiviral immunity (IFN‐λ, IFN‐β), immune regulation (MCP1, Eotaxin, TNFR1, TNFR2, IP‐10, PD‐L1, TSLP), endothelial dysfunction (VCAM‐1), inflammation (Procalcitonin, IL‐10), coagulation (P‐selectin), axonal injury (Tau), and tissue repair (β‐NGF) pathways (Figure S2).

Based on the observed associations between Cluster 6 and elevated markers of coagulation and prior published reports linking PASC to altered coagulopathy and complement activation, specifically component 3 of the complement pathway, we performed an exploratory protein–protein interaction analysis using STRINGdb to explore possible associations between the specific biomarkers within the inflamed clusters (clusters 4, 5, and 6) and component 3 of the complement pathway. This analysis revealed that the overexpressed biomarkers of our three inflamed clusters were linked to component 3 of the complement pathway, with Cluster 6 having the strongest interaction (Figure 4).

**Figure 4:**
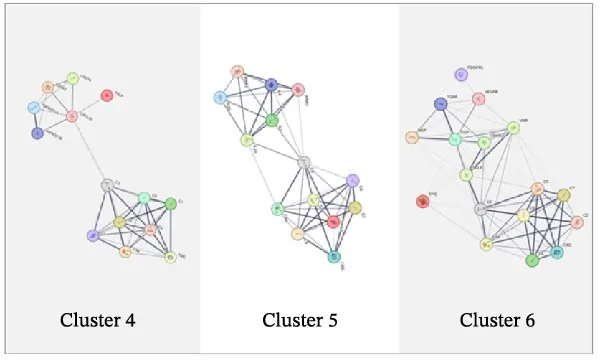
Network analysis—interaction between complement 3 and key biomarkers in clusters 4, 5, and 6. Protein–protein interaction network based on known interactions available through the STRING.

To determine whether biomarker clusters predicted recovery from PASC, we conducted a follow‐up assessment among the 356 participants. Of these, 109 (30.6%) were directly contactable by phone, while the remaining participants were assessed through a combination of documented medical consultation and review of electronic medical records. Overall, 196 (55.1%) participants reported recovery, with recovery rates exceeding 50% in every cluster except clusters 4 (48%) and 6 (47%).

Consistent with this finding, in the predefined clinical phenotypes, we observed a higher recovery rate in the “Less Symptomatic” clinical group (66%) when compared to the cardiorespiratory and musculoskeletal clinical phenotypes (Table S2). In analysis adjusted for age, sex at birth, ethnicity, BMI, smoking status, alcohol, using the “Less Symptomatic” clinical group as a reference, both the cardiorespiratory (0.539 [95% CI 0.290, 1.000]) and musculoskeletal (0.331 [95% CI 0.172, 0.637]) groups were significantly less likely to recover.

When examining individual symptoms and associations with recovery, participants in the non‐recovered group exhibited a significantly higher prevalence of fatigue, SOB, chest pain, poor concentration, and myalgia compared to the recovered group, all *p* < 0.05.

## 4. Discussion

This study used clustering approaches to stratify people with PASC by their biological phenotype into six distinct biological clusters, each with unique clinical and biological characteristics. Three of these clusters were associated with higher markers of inflammation, tissue damage and coagulation, greater likelihood of concurrent comorbidities, and linked to respiratory symptoms of PASC and lower recovery rates. To our knowledge, this is one of the first studies to stratify people with PASC into distinct clusters based on their biological phenotypes. Of particular interest, elevated levels of markers of tissue damage, coagulation, and axonal injury were observed in one of the clusters (Cluster 6), which was also significantly associated with more cardiac and respiratory symptoms of PASC in fully adjusted analysis. Notably, these biological clusters were derived independently of, and were not predicted by, the pre‐existing symptom‐derived clinical phenotypes, which did not differ in any of the 57 measured biomarkers. This indicates that clinical presentation alone is insufficient to resolve the biological heterogeneity of PASC and that it is biomarker‐derived stratification, rather than symptom‐based classification, that identifies clinically meaningful inflammatory endotypes. These findings suggest that PASC is underpinned by distinct, biologically‐defined inflammatory endotypes that are not captured by symptom‐based classification alone and point to the need for a more personalized approach, grounded in biomarker profiling rather than clinical presentation, to investigate potential, targeted treatments.

The identification of six distinct clusters likely reflects a combination of separate underlying disease mechanisms, different temporal stages of recovery, as well as reflecting host biological susceptibility. For example, the overlap between inflammatory, endothelial, and coagulation pathways observed across clusters 4, 5, and 6 indicates that these endotypes reflect persistent innate immune activation coupled to vascular dysfunction, consistent with emerging long COVID models. However, the distinct biomarker signatures suggest different triggering mechanisms: Cluster 5’s antiviral signature (IFN‐α2, IFN‐β) may represent ongoing viral antigen‐driven responses, Cluster 4’s pattern (TNFR1, TNFR2, IL‐1Ra) appears consistent with host metabolic inflammation seen in older individuals with more comorbidities, while Cluster 6’s neuroimmune profile suggests a distinct pathway involving neuroinflammation and vascular dysfunction. Whether these clusters represent stable endotypes or dynamic states that evolve over time cannot be determined from our cross‐sectional design and requires longitudinal investigation before these inflammatory clusters can be considered stable, clinically meaningful biomarkers of the PASC subtype.

The elevated levels of tissue damage and coagulation biomarkers observed in inflamed clusters align with previous studies implicating hypercoagulability and endothelial dysfunction in PASC [26]. A recent in‐depth blood proteomic analysis in 113 individuals with confirmed COVID‐19, 40 of whom had PASC, showed proteomic patterns reflecting ongoing thrombo‐inflammation, suggesting that people with PASC may still experience vascular inflammation and potentially increased risk of blood clot formation long after the acute SARS‐CoV‐2 infection [22]. It is worth noting that Cluster 6 was also characterized by elevated levels of markers linked to tissue damage and coagulation. Research on PASC has highlighted persistent coagulation abnormalities, often referred to as thrombo‐inflammation, which play a key role in the pathophysiology of this condition. These abnormalities may contribute to prolonged symptoms, such as fatigue, dyspnea, cognitive impairment, and muscle pain. Proposed mechanisms to explain these abnormalities include complement dysregulation, protein C system dysfunction, and the formation of NETs (neutrophil extracellular traps) [27]. Consistent with this, in the three inflamed clusters, we observed links to component 3 of the complement pathway, which is consistent with previous published findings [22]. However, it is important to emphasize that our network analysis, based on STRING database protein–protein interaction predictions, remains exploratory in nature. While these bioinformatic associations highlight potential functional relationships between complement activation, coagulation cascades, and inflammatory mediators, they are insufficient to establish mechanistic causality and require validation in functional studies.

While the absence of a non‐PASC control group, including both healthy controls and recovered COVID‐19 individuals without persistent symptoms, in this study limits our ability to definitively attribute the observed inflammatory clusters to PASC itself, the temporal association between COVID‐19 infection and persistent symptoms in our cohort suggests that these inflammatory profiles may be at least partially driven by postacute sequelae rather than solely by pre‐existing conditions such as advancing age and BMI. However, without these comparator groups, we cannot exclude that some of the observed biomarker signatures reflect persistent postinfectious immune remodeling common to COVID‐19 convalescence in general rather than mechanisms unique to PASC. Future studies including recovered individuals without PASC are essential to establish the specificity of these inflammatory endotypes.

Of the six clusters, Cluster 6 displayed the most intriguing inflammatory pattern. Elevated levels of five biomarkers involved in the axonal injury pathway, including S100A9, S100B, UCHL1, TAU, and ENO2, were observed in Cluster 6, supporting findings from recent studies pointing to neurological involvement in PASC [7, 28]. These biomarkers are frequently associated with neuronal damage and neuroinflammation in chronic neurological diseases. Although we have assigned these markers to an axonal injury pathway, these markers are not exclusively specific to CNS injury and may also reflect systemic inflammatory processes, blood‐brain barrier disruption, or vascular dysfunction affecting neural tissues. Future studies incorporating neuroimaging or cerebrospinal fluid analysis will be necessary to distinguish between these mechanisms and determine the clinical significance of these biomarker changes for long‐term neurological outcomes.

To address the concern that observed inflammatory signatures may be driven by metabolic inflammation, we examined the robustness of cluster‐clinical associations after adjustment for age and BMI. Forest plots (Figure 3) demonstrate that associations between biomarker‐derived clusters and cardiorespiratory symptoms remained significant in fully adjusted models, indicating that the identified inflammatory endotypes are not entirely attributable to age‐related or metabolic factors.

Although other studies have also observed alterations in biomarkers associated with axonal injury in acute COVID and PASC [28, 29], they focused on neurofilament light chain (NFL) and glial fibrillary acidic protein (GFAP), in contrast to the expanded panel of markers examined in our analysis. Both plasma NFL and GFAP were higher in acute COVID and associated with neurological symptoms, and GFAP levels were higher in people with PASC. However, there were no associations with these markers and neurological symptoms in PASC. Interestingly, within our analysis, although NFL and GFAP did not discriminate between clusters, within the inflamed clusters, the prevalence of reported poor concentration was highest in Cluster 6 (47%) compared to Cluster 4 (40%) and Cluster 5 (19%). The observation that both neuroinflammation and elevations in axonal injury biomarkers have been linked to neurodegenerative conditions like Alzheimer’s and Parkinson’s highlights the need for more research on the long‐term impact of elevations in these biomarkers in people with PASC [30, 31].

Cluster 4 showed elevations in biomarkers mapping to innate immune activation, including TNFR1, TNFR2, IL‐1Ra, and CD163. This group also included people of older age (median [IQR] age 57 [49–65] years) and, along with Cluster 6 participants, a higher median BMI (>30 kg/m^2^) consistent with obesity. Both factors are associated with increased inflammation in the general population [32], and both the IL‐1 and TNF pathways have been associated with inflammation in obesity [33]. Cluster 4 also had a higher prevalence of comorbidities such as hypertension and obesity, reflecting a possible association between this biological phenotype and metabolic syndrome. Elevated levels of these proinflammatory markers are consistent with reports of chronic inflammation observed in recent studies on PASC participants [7], suggesting ongoing immune activation and systemic inflammation contributing to persistent symptoms observed in PASC [34]. However, an alternative explanation may be that Cluster 4 simply reflects the expected proinflammatory pattern linked to old age and obesity rather than a pattern specific to PASC. The high prevalence of metabolically related comorbidities in this cluster would support this hypothesis. This high‐inflammatory cluster 4 is indeed characterized by cardiometabolic comorbidities and elevated BMI, which are known contributors to systemic inflammation. Nevertheless, the persistence of symptoms and the specific biomarker profiles observed in these patients warrant further investigation to determine whether PASC exacerbates or modifies pre‐existing inflammatory pathways.

Highlighting this interplay between age, cardiopulmonary comorbidities, and COVID‐19 outcomes, a recent study showed older individuals with pre‐existing cardiac and pulmonary conditions to be at heightened risk for both severe COVID‐19 and prolonged recovery [35]. These findings align with our observation that patients in proinflammatory Cluster 4 exhibited higher prevalence of cardiometabolic comorbidities and elevated BMI, suggesting that PASC may exacerbate or interact with pre‐existing inflammatory states associated with aging and chronic disease.

In contrast, Cluster 5 had higher levels of antiviral biomarkers (IFN‐α2 and IFN‐β), suggesting an antiviral persistent immune response to infection, coupled with higher levels of anti‐inflammatory cytokines (IL‐2, IL‐4, and IL‐10). Immune activation driven by persistence of viral antigens or reactivation of latent/dormant viruses has been described in several viral infections and has been proposed as a possible explanation for PASC [36–38]. Several mechanisms could explain the sustained antiviral signatures in Cluster 5: (i) persistent SARS‐CoV‐2 reservoirs in immunologically privileged tissues providing ongoing antigenic stimulation; (ii) persistent viral antigen presentation without active replication; or (iii) reactivation of latent herpesviruses triggered by SARS‐CoV‐2 infection [39]. Measurement of direct viral persistence was beyond the scope of this study, and whether the inflammatory pattern in Cluster 5 is caused by viral persistence requires further investigation using sensitive viral detection methods alongside longitudinal immune profiling.

Although vaccination status has been reported to influence COVID‐19 disease severity and pulmonary involvement [40], we did not observe significant correlations between vaccination status and the high inflammatory biomarker levels in our cohort. This suggests that the inflammatory clusters identified may develop independently of vaccination history, though further investigation in more diverse vaccination cohorts is warranted.

Out of all the reported symptoms of PASC, palpitations were associated with both clinical/demographic characteristics (older age and more severe initial COVID‐19) as well as, somewhat unexpectedly, lower levels of inflammatory biomarkers (Figure S2). This has also been observed in other studies [4, 25]. These observations would suggest that the etiology of palpitations, a common reported symptom of PASC, may not have an underlying inflammatory cause and might require alternative management strategies other than modifying host inflammatory responses.

Host inflammatory patterns were also associated with recovery in PASC. While the overall reported recovery rate was 55%, clusters 4 (48%) and 6 (47%) exhibited the lowest recovery percentage of all clusters. However, this difference was not statistically significant. Interestingly, participants reporting fewer symptoms (belonging to the “Less Symptomatic” clinical phenotype) seemed to have significantly better recovery independent of factors such as demographics, BMI, smoking status, and comorbidities. This pattern warrants further investigation in future research.

Participants were enrolled at 4–10 weeks postinfection, representing the early postacute phase rather than established chronic PASC (typically >12 weeks). During this transitional period, immune remodeling is active, and some degree of inflammatory biomarker elevation may reflect normal, albeit delayed, recovery from acute infection rather than stable, chronic, persistent inflammatory states. Comparative longitudinal studies with repeated sampling are needed to determine whether these inflammatory endotypes persist into chronic PASC or resolve during recovery and which early biomarker signatures predict progression to chronic disease versus eventual recovery.

Our cohort comprised 74.4% female participants, consistent with the female predominance observed in PASC cohorts [8]. Female participants were distributed similarly across all clusters (*p* = 0.7), suggesting that sex did not significantly influence cluster membership in our analysis. However, sex differences in immune responses to viral infections are well documented, and future research examining sex‐specific inflammatory endotypes would be valuable for determining whether the biological mechanisms underlying these clusters manifest differently in male versus female patients.

The identification of distinct inflammatory clusters in our PASC cohort represents a first step toward a precision medicine approach to clinical management of PASC. While further validation is required, this clustering approach may inform targeted therapeutic strategies tailored to specific inflammatory profiles. Future longitudinal studies with appropriate control groups could assess cluster stability over time and determine whether specific biomarker signatures can predict clinical outcomes, treatment response, or disease progression. Such insights could ultimately guide clinicians in stratifying patients for precision medicine approaches in PASC care.

## 5. Limitations and Perspectives

Our study has some limitations. The cross‐sectional design limits our ability to assess causality. In addition, as participants were sampled at various timepoints postonset of initial acute COVID‐19, any dynamic change in host inflammatory responses could not be assessed in a standardized manner. In addition, data on the use of corticosteroids, immunomodulatory therapies, and anticoagulants were not systematically recorded, and we were unable to assess their impact on the observed biomarker profiles.

The lack of a non‐PASC control group also limits interpretation as to which inflammatory patterns are specific to PASC and which could also be explained by non‐COVID features. As previously discussed, the inflammatory pattern observed in Cluster 4 could be explained by older age and higher BMI rather than by PASC specifically. Our cross‐sectional design also prevents us from addressing important outstanding questions regarding longitudinal relationships between inflammation and PASC, including whether the clusters represent stable endotypes or dynamic states that evolve over time and how they relate to the persistence of symptoms or recovery from PASC. Longitudinal studies with serial biomarker measurements are needed to identify which inflammatory signatures predict persistent symptoms versus eventual recovery. Furthermore, participants were enrolled at 4–10 weeks postinfection, representing the early postacute phase rather than established chronic PASC (typically >12 weeks). The inflammatory clusters identified may therefore preferentially represent early postacute immunopathology rather than stable chronic inflammatory states. Although we conducted a targeted laboratory analysis, we were limited in not being able to assess viral persistence, and there may be other, unmeasured biomarkers that could provide additional insights into pathogenesis. In addition, our interpretation of linking changes in biomarkers to specific biological functions (e.g., axonal injury) may be limited by the fact that some biomarkers may have two or more functions.

Due to the exploratory nature of this study and the inherent correlation between inflammatory biomarkers, we did not apply formal correction for multiple testing in biomarker‐symptom association analyses. Consequently, findings should be interpreted cautiously, with emphasis on overall biological patterns and effect sizes rather than isolated *p*‐values. Future confirmatory studies with larger sample sizes should employ appropriate multiple‐testing corrections.

The use of a mixed approach for recovery assessment, combining telephone interviews and medical records, may have introduced some heterogeneity in the data collection. However, this pragmatic strategy was necessary given the challenges of participant follow‐up in a real‐world cohort study. Future longitudinal studies, including appropriate non‐PASC control groups, could help to overcome some of these limitations.

In conclusion, six distinct inflammatory clusters were identified within people with PASC, three of which showed elevated biomarkers related to tissue damage and repair, coagulation, axonal injury, and antiviral immune responses and were associated with component 3 of the complement pathway. This is one of the first studies linking specific biological pathways, notably axonal injury and thrombo‐inflammation, with the development of PASC. These results provide a better understanding of the underlying mechanisms that contribute to the development of PASC, highlighting the importance of recognizing these biological differences for potential targeted treatments. Future longitudinal studies incorporating matched control groups, including both healthy individuals and those with similar comorbidities but without PASC, will be essential to confirm whether the inflammatory clusters identified here are specific to PASC or represent an amplification of baseline inflammatory conditions.

## Author Contributions

Ninon Marmont, Alejandro Abner Garcia Leon, and Patrick W. G. Mallon participated in conceptualization, formal analysis, project administration, supervision, writing the original draft, and review and editing of the final draft. Ninon Marmont and Alejandro Abner Garcia Leon participated in data curation. Alejandro Abner Garcia Leon, Fiorina Rigonat, Polina Smolovyk, and Patrick W. G. Mallon contributed to funding acquisition. Ninon Marmont, Alejandro Abner Garcia Leon, Grace Kenny, Eoghan de Barra, Eoin Feeney, Kathleen McCann, Mary Horgan, Obada Yousif, Andrew Conroy, Corinna Sadlier, Aoife G. Cotter, Jane A. O’Halloran, Stefano Savinelli, Fiorina Rigonat, Polina Smolovyk, Colette Gaillard, and Patrick W. G. Mallon participated in investigation and care management. All authors were involved in drafting the manuscript.

## Funding

Ninon Marmont was funded through a fellowship from the Centre for Experimental Pathogen Host Research core funding during the study.

## Ethics Statement

The All‐Ireland Infectious Diseases Cohort study was approved in line with national and European regulations on health research by the St Vincent’s Hospital Research Ethics Committee and the National Research Ethics Committee for COVID‐19 in Ireland (Study Reference RS20‐033). Participants provided written informed consent for the collection of data on demographics, clinical characteristics, and investigations undertaken as part of routine care.

## Conflicts of Interest

The authors declare no conflicts of interest.

## Supporting Information

Additional supporting information can be found online in the Supporting Information section.

## Supporting information


**Supporting Information** Table S1: Biomarkers classified by pathways and methods used. Table S2: Recovery assessment and baseline symptoms. Table S3 (A–H): Biological pathways and expected roles of the 57‐biomarker panel. Figure S1 (A–H): Expression of biomarkers belonging to each pathway (axonal injury, inflammation, antiviral immune responses, immune regulation/innate immune activation, tissue damage and repair, endothelial dysfunction, coagulation, and microbial translocation) through the six clusters. Each data point represents a single biomarker measurement from individual participants (Cluster 1 [*n* = 4], Cluster 2 [*n* = 108], Cluster 3 [*n* = 140], Cluster 4 [*n* = 42], Cluster 5 [*n* = 43], and Cluster 6 [*n* = 19]). No technical replicates were performed. Statistical significance was assessed using Kruskal–Wallis tests followed by Dunn’s multiple comparison tests with significance set at *p* < 0.05. Figure S2: Heatmap showing the correlation between the symptoms and the level of biomarkers.

## Data Availability

The data that support the findings of this study are available upon request from the corresponding author. The data are not publicly available due to privacy or ethical restrictions. This article does not report the original code. Any additional information required to reanalyze the data reported in this article is available from the lead contact upon request.
