# Supplementary material for: Characterizing Host Inflammatory Bioprofiles in People With Postacute Sequelae SARS‐CoV‐2 Infection (PASC)
Source: J Immunol Res. 2026 Sep 29;2026:6436047. doi: 10.1155/jimr/6436047 (PMC13621821; doi:10.1155/jimr/6436047)
Supplement: Supplementary file 1 [file JIMR-2026-6436047-s001.docx]

# Supplementary Material

## Supplementary material – Table 1:

## Table S1 - Biomarkers classified by pathways and methods used

| Biological Pathway | Luminex - Multiplex (39 Biomarkers) | MSD - Multiplex  (16 Biomarkers) | Single Plex  (2 Biomarkers) |
| --- | --- | --- | --- |
| Inflammation | TNF-α, Procalcitonin, IL-6, IL-1β, IL-12, IL-17, IL-8 | IL-10, IL-2, IL-4, IL-7 | / |
| Antiviral immunity | IFN-γ, IFN-λ2 (also known as IL-28A), IFN-λ3 (also known as IL-28B) | IFN-α2, IFN-β, IFN-λ1 (also known as IL-29) | / |
| Endothelial dysfunction | ICAM-1, VCAM-1 | / | / |
| Tissue repair | VEGF, EGF, PDGF-AA, TGF-α, β-NGF, vWF | / | / |
| Coagulation | D-dimer, CD40L, P-selectin, TPO | / | / |
| Microbial translocation | / | I-FABP, LBP | Histamine, β-D-Glucan |
| Innate immune activation / Immune regulation | CD163, MCP-1, MIP-1α, IL-18, IP-10, GM-CSF, MCP-3, TNFR1, TNFR2, IL-1Ra, PD-L1 | TSLP, Eotaxin, IL-33, CD14 | / |
| Axonal injury | UCHL-1, MAPT, S100A9, S100A8, S100B, ENO2 | NF-L, GFAP, MBP | / |

## Supplementary material – Table 2:

Table S2 – Recovery Assessment & Baseline Symptoms

Supplementary material – Table 3:

Table S3.A. Expected Biological Role of Biomarkers Belonging to the Inflammation Pathway

| **Biomarker** | **Assay** | **Expected Biological Role** |
| --- | --- | --- |
| TNF-α | Luminex | Pro-inflammatory cytokine; mediates acute-phase response and immune cell activation |
| Procalcitonin | Luminex | Marker of systemic inflammation, classically associated with bacterial infection |
| IL-6 | Luminex | Key pro-inflammatory cytokine driving acute-phase response; elevated in severe COVID-19 |
| IL-1β | Luminex | Pro-inflammatory cytokine; activated via the inflammasome pathway |
| IL-12 | Luminex | Promotes Th1 differentiation; bridges innate and adaptive immunity |
| IL-17 | Luminex | Pro-inflammatory cytokine; mediates neutrophil recruitment (Th17 pathway) |
| IL-8 (CXCL8) | Luminex | Chemokine; primary neutrophil chemoattractant |
| IL-10 | MSD | Anti-inflammatory/regulatory cytokine; dampens immune activation |
| IL-2 | MSD | T-cell growth factor; promotes T-cell proliferation |
| IL-4 | MSD | Th2 cytokine; promotes humoral (antibody-mediated) immunity |
| IL-7 | MSD | Supports lymphocyte development and homeostasis |

Table S3.B. Expected Biological Role of Biomarkers Belonging to the Antiviral Immunity Pathway

| **Biomarker** | **Assay** | **Expected Biological Role** |
| --- | --- | --- |
| IFN-γ | Luminex | Type II interferon; activates macrophages, antiviral/antimicrobial defense |
| IFN-α2 (IL-28A) | Luminex | Type I/III interferon; antiviral response |
| IFN-α3 (IL-28B) | Luminex | Type III interferon; antiviral mucosal immunity |
| IFN-α2 | MSD | Type I interferon; early antiviral response |
| IFN-β | MSD | Type I interferon; early antiviral response |
| IFN-λ1 (IL-29) | MSD | Type III interferon; antiviral defense at mucosal surfaces |

Table S3.C. Expected Biological Role of Biomarkers Belonging to the Endothelial Dysfunction Pathway

| **Biomarker** | **Assay** | **Expected Biological Role** |
| --- | --- | --- |
| ICAM-1 | Luminex | Adhesion molecule; mediates leukocyte-endothelial adhesion, marker of endothelial activation |
| VCAM-1 | Luminex | Adhesion molecule; marker of vascular inflammation |

Table S3.D. Expected Biological Role of Biomarkers Belonging to the Tissue Repair Pathway

| **Biomarker** | **Assay** | **Expected Biological Role** |
| --- | --- | --- |
| VEGF | Luminex | Promotes angiogenesis and vascular repair |
| EGF | Luminex | Promotes epithelial cell proliferation and tissue repair |
| PDGF-AA | Luminex | Promotes fibroblast proliferation and wound healing |
| TGF-α | Luminex | Growth factor supporting epithelial repair |
| β-NGF | Luminex | Neurotrophic factor; supports nerve/tissue repair |
| VWF | Luminex | Marker of endothelial activation and platelet adhesion |

Table S3.E. Expected Biological Role of Biomarkers Belonging to the Coagulation Pathway

| **Biomarker** | **Assay** | **Expected Biological Role** |
| --- | --- | --- |
| D-dimer | Luminex | Fibrin degradation product; marker of coagulation activation/thrombosis |
| CD40L | Luminex | Platelet activation marker; links coagulation and inflammation |
| P-selectin | Luminex | Platelet/endothelial activation marker; mediates leukocyte adhesion |
| TPO | Luminex | Regulates platelet production |

Table S3.F. Expected Biological Role of Biomarkers Belonging to the Microbial Translocation Pathway

| **Biomarker** | **Assay** | **Expected Biological Role** |
| --- | --- | --- |
| I-FABP | MSD | Marker of intestinal epithelial damage / gut barrier dysfunction |
| LBP | MSD | Lipopolysaccharide-binding protein; marker of bacterial translocation |
| Histamine | Single Plex | Mediator of immune/allergic response; associated with gut permeability |
| β-D-Glucan | Single Plex | Fungal cell wall component; marker of microbial translocation |

Table S3.G. Expected Biological Role of Biomarkers Belonging to the Innate Immune Activation and Immune Regulation Pathway

| **Biomarker** | **Assay** | **Expected Biological Role** |
| --- | --- | --- |
| CD163 | Luminex | Macrophage activation marker (scavenger receptor) |
| MCP-1 (CCL2) | Luminex | Chemokine; monocyte chemoattractant |
| MIP-1α (CCL3) | Luminex | Chemokine; recruits monocytes/macrophages |
| IL-18 | Luminex | Pro-inflammatory cytokine; activates NK and T cells |
| IP-10 (CXCL10) | Luminex | Chemokine; T cell recruitment, marker of interferon response |
| GM-CSF | Luminex | Promotes myeloid cell differentiation and activation |
| MCP-3 (CCL7) | Luminex | Chemokine; monocyte/macrophage recruitment |
| TNFR1 | Luminex | Soluble TNF receptor; marker of TNF pathway activation |
| TNFR2 | Luminex | Soluble TNF receptor; marker of chronic inflammation |
| IL-1Ra | Luminex | IL-1 receptor antagonist; anti-inflammatory regulatory marker |
| PD-L1 | Luminex | Immune checkpoint molecule; marker of immune regulation/exhaustion |
| TSLP | MSD | Epithelial-derived cytokine; promotes Th2 response |
| Eotaxin (CCL11) | MSD | Chemokine; eosinophil chemoattractant |
| IL-33 | MSD | Alarmin; activates innate lymphoid cells and mast cells |
| CD14 | MSD | Monocyte activation marker; LPS co-receptor |

Table S3.H. Expected Biological Role of Biomarkers Belonging to the Axonal Injury Pathway

| **Biomarker** | **Assay** | **Expected Biological Role** |
| --- | --- | --- |
| UCHL-1 | Luminex | Neuronal marker; indicates neuronal cell body damage |
| MAPT (Tau) | Luminex | Marker of axonal injury and neurodegeneration |
| S100A9 | Luminex | Damage-associated molecular pattern (DAMP); marker of inflammation and tissue damage — not CNS-specific |
| S100A8 | Luminex | Neutrophil-derived DAMP; marker of systemic inflammation — not CNS-specific |
| S100B | Luminex | Marker of glial/astrocyte damage; may also reflect systemic vascular injury |
| ENO2 (NSE) | Luminex | Neuron-specific enolase; marker of neuronal injury |
| NF-L | MSD | Neurofilament light chain; marker of axonal damage |
| GFAP | MSD | Glial fibrillary acidic protein; marker of astrocyte injury |
| MBP | MSD | Myelin basic protein; marker of demyelination and white matter injury |

## Supplementary material – Figure 1:

Figure S1.A. Levels of Immune Activation Biomarkers by Clusters.

Figure S1.B. Levels of Biomarkers of Inflammation by Clusters.

Figure S1.C. Levels of inflammation and Antiviral immunity Biomarkers by Clusters.

Figure S1.D. Levels of Tissue repair Biomarkers by Clusters

Figure S1.E. Levels of Axonal injury Biomarkers by Clusters

Figure S1.F. Levels of Coagulation Biomarkers by Clusters

Figure S1.G. - Levels of Endothelial Biomarkers by Clusters

Figure S1.H. Levels of Microbial translocation Biomarkers by Clusters

Supplementary material – Figure 2:

Figure S2 - Heatmap showing the correlation between the symptoms and the level of biomarkers
